# Machine Learning Approaches for Epidemiological Investigations of Food-Borne Disease Outbreaks

**DOI:** 10.3389/fmicb.2019.01722

**Published:** 2019-08-06

**Authors:** Baiba Vilne, Irēna Meistere, Lelde Grantiņa-Ieviņa, Juris Ķibilds

**Affiliations:** ^1^Institute of Food Safety, Animal Health and Environment—“BIOR”, Riga, Latvia; ^2^SIA net-OMICS, Riga, Latvia

**Keywords:** machine learning, food-borne disease, outbreaks, bacterial WGS, bioinformatics analysis pipeline

## Abstract

Foodborne diseases (FBDs) are infections of the gastrointestinal tract caused by foodborne pathogens (FBPs) such as bacteria [*Salmonella, Listeria monocytogenes* and Shiga toxin-producing *E. coli* (STEC)] and several viruses, but also parasites and some fungi. Artificial intelligence (AI) and its sub-discipline machine learning (ML) are re-emerging and gaining an ever increasing popularity in the scientific community and industry, and could lead to actionable knowledge in diverse ranges of sectors including epidemiological investigations of FBD outbreaks and antimicrobial resistance (AMR). As genotyping using whole-genome sequencing (WGS) is becoming more accessible and affordable, it is increasingly used as a routine tool for the detection of pathogens, and has the potential to differentiate between outbreak strains that are closely related, identify virulence/resistance genes and provide improved understanding of transmission events within hours to days. In most cases, the computational pipeline of WGS data analysis can be divided into four (though, not necessarily consecutive) major steps: *de novo* genome assembly, genome characterization, comparative genomics, and inference of phylogeny or phylogenomics. In each step, ML could be used to increase the speed and potentially the accuracy (provided increasing amounts of high-quality input data) of identification of the source of ongoing outbreaks, leading to more efficient treatment and prevention of additional cases. In this review, we explore whether ML or any other form of AI algorithms have already been proposed for the respective tasks and compare those with mechanistic model-based approaches.

## 1. Introduction

Foodborne diseases (FBDs) are infections of the gastrointestinal tract caused by foodborne pathogens (FBPs) such as bacteria and several viruses, but also parasites and some fungi. *Salmonella, Listeria monocytogenes* and Shiga toxin-producing *Escherichia coli* (STEC) are some of the most important bacterial FBPs (Sekse et al., 2017), causing the most outbreaks and the largest number of sporadic cases with severe illness or even fatal outcome (EFSA, 2015; Sekse et al., 2017). *Salmonella* infections affect people at all ages and the main food sources of infection typically include ready-to-eat foods, eggs, swine and poultry. *L. monocytogenes* infections mostly affect elderly people, as well as immunocompromised patients and pregnant women, and display high mortality rates. Common food sources of *L. monocytogenes* include ready-to-eat foods such as smoked fish and soft cheeses. STEC has been associated with severe complications, e.g., acute kidney failure, often affecting elderly and immunocompromised people, and also small children. The main food sources of STEC infections are bovine meat, followed by vegetables and juice (EFSA, 2015).

Whole-genome sequencing (WGS) is becoming more accessible and affordable as a routine approach for early detection of FBD outbreaks (Buultjens et al., 2017; Sekse et al., 2017). WGS captures the entire genome within hours to days and has the potential to differentiate between outbreak strains that are closely related, identify virulence/resistance genes and provide improved understanding of transmission events (Quainoo et al., 2017; Andersen and Hoorfar, 2018). Moreover, third-generation sequencing technologies such as Oxford Nanopore (ONT) sequencing and PacBio Single Molecule, Real-Time (SMRT), which allow the generation of ultra-long (up to 300 kb) reads, are well suited to assemble reference genomes from outbreak strains *de novo*, potentially contributing to more precise taxonomic assignment, while offering increased detection speed and relatively decreasing costs, as, in comparison to Illumina short-read sequencing, both technologies are still three and almost seven times more expensive, respectively (Brown et al., 2017; Sekse et al., 2017; Nicola De Maio, 2019). Several *proof-of-concept* studies have demonstrated the superiority of WGS over traditional typing methods for a range of high priority food-borne pathogens, e.g., *Salmonella enterica, Listeria monocytogenes, Campylobacter species* and STEC (Kanamori et al., 2015; Quick et al., 2015; Moran-Gilad, 2017). Large initiatives have emerged to investigate the options of replacing conventional methods with WGS for outbreak investigations. Two such examples include the ENGAGE (Establishing Next Generation sequencing Ability for Genomic analysis in Europe) (Hendriksen et al., 2018) and INNUENDO projects (Llarena et al., 2018), focusing on the idevelopment of dedicated analytical platforms and standardized analysis pipelines, e.g., for *E. coli* and different *Salmonella* spp. serotypes (Hendriksen et al., 2018).

In the era of Big Data, as the volume and complexity of data increases steadily, artificial intelligence (AI) and its sub-discipline machine learning (ML) are re-emerging and gaining an ever increasing popularity in the scientific community and industry (Ching et al., 2018). While mechanistic model-based approaches aim at constructing simplified mathematical formulations, i.e., hypothesis, of causal mechanisms by carefully observating, analyzing and trying to understand the complexity of the respective phenomenon (Baker et al., 2018), machine learning (ML) algorithms use large-scale datasets to extract meaningful patterns (i.e., “learn”) and use this “knowledge” to make predictions on other data (Alkema et al., 2016). Moreover, ML can be done in a unsupervised manner by exploring and detecting patterns within the data or in a supervised manner by classifying, predicting and explaining (Tebani et al., 2016). Unsupervised ML techniques involve well-known and widely used methods such as principal component analysis (PCA) and k-means clustering (Tebani et al., 2016). PCA is a dimensionality reduction method, transforming a large set of variables into a smaller set, while preserving as much information as possible (Hotelling, 1933), whereas k-means clustering groups similar data points together in a fixed number (k) of clusters and tries to discover their underlying patterns (Hartigan and Wong, 1979). In life sciences, some frequently used supervised ML strategies have been Random Forest (RF), Support Vector Machines (SVM), Naive Bayes (NB), and Artificial Neural Networks (Lai et al., 2016). RF alorithm randomly selects a subset from the training data to construct an ensemble of decision tree predictors to aggregate the predictions, thus lowering the variance (Breiman, 1996). SVM represent a pattern classification technique, which is based on the idea of transforming the original data that is not linearly separable to a higher dimensional space and finding a hyperplane separating the data into classes (Boser et al., 1992). NB represents a probabilistic algorithm that uses the probability theory and Bayes' Theorem in conjunction with prior knowledge to calculate the probability of each feature to belong to each of the classes and then outputs the class with the highest probability (Devroye et al., 2013). Finally, ANNs are graph computing models, which, at least to some extent, should mimic the functioning of the human brain, hence its computing units are called neurons and are interconnected for passing information to each other. Moreover, networks of neurons are additionally organized in layers. The first one is an input layer, receiving the training data. This is followed by several hidden layers. The last one is an output layer, which performs the actual prediction of the class (Kruse et al., 2016).

Global multi-disciplinary initiatives like One Health (OH) (http://www.onehealthinitiative.com/), aiming toward optimizing the health of people, animals and the environment, would greatly profit from such approaches, as multiple complex challenges need to be addressed, including the maintenance of a safe food and water supply for a growing human population. Considering the current ease with which people and animals or animal products can be transported around the globe, the forefront issues of OH are clearly related to spread of emerging infectious diseases and antimicrobial resistance (AMR) (Gibbs, 2014). Especially, outbreaks caused by multi-drug-resistant bacteria are an urgent and growing global public health threat (CDC, 2013; WHO, 2014). Effective management protocols must be in place, as quick identification leads to faster and more precisely targeted treatment (Quainoo et al., 2017).

ML strategies have already been used for microbial diagnostics in diverse contexts, including (i) taxonomic grouping of metagenomics data (Sedlar et al., 2017; Afify and Al-Masni, 2018); (ii) classification of *L. monocytogenes* persistence in retail delicatessen environments (Vangay et al., 2014); (iii) phenotype prediction of bacterial strains based on presence/absence of particular genes (i.e., gene-trait matching) (Dutilh et al., 2013; Alkema et al., 2016; Farrell et al., 2018); (iv) to identify strains that demonstrate a higher probability to cause severe diseases (Wheeler et al., 2018); (v) to predict the host range of pathogens (Lupolova et al., 2017), e.g., identifying their signatures of host adaptation (Wheeler et al., 2018); and (vi) to predict the antimicrobial resistance potential of different *E. coli* strains (Her and Wu, 2018) or from different sources (Li et al., 2018).

**Figure 1:**
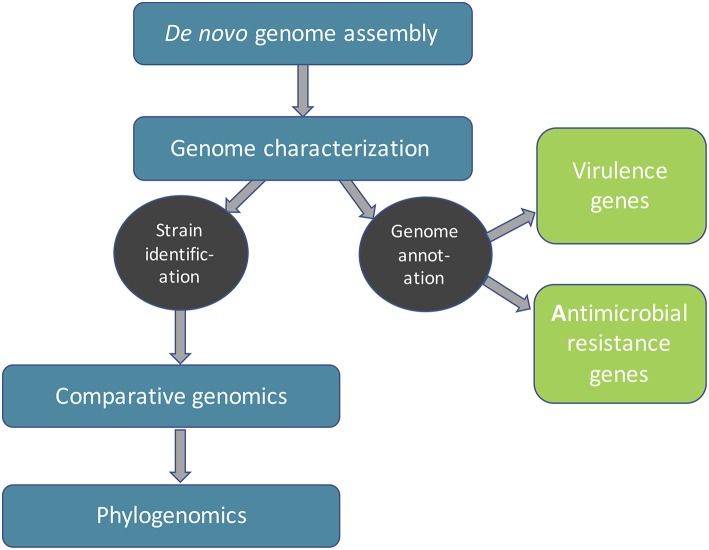
An overview of an example bacterial sequence data analysis workflow.

The WGS data analysis pipeline can be generally divided into four major steps (Figure 1): *de novo* genome assembly, genome characterization, comparative genomics and inference of phylogeny or phylogenomics (Quainoo et al., 2017). However, these steps are not necessarily consecutive, depending on the objectives of the study. ML could be used in any of these analyses to increase the speed and potentially accuracy (provided increasing amounts of high-quality input data). In this review, we aim to explore whether ML algorithms have already been proposed for the respective task and compare those algorithms with mechanistic model-based approaches (see Table 1 for an overview). We mainly focus on single-genome short-read (Illumina) bacterial WGS; however in cases where, to the best of our knowledge, no ML algorithms have been reported for the respective task, we also briefly touch upon ML algorithms dedicated to ultra-long read technologies, 16S metataxonomics and shotgun metagenomics, as these approaches may find future applications in FBD outbreaks. Currently, the starting point for any FBD outbreak investigation involving strain typing is access to isolates, which may be difficult to obtain or are often even unavailable. Moreover, most food samples are complex, harboring composite microbial communities. In this regard, metagenomic approaches would allow one to capture the full spectrum of microbes in foods entirely without prior need for culturing and isolation, allowing also the detection of “viable but not cultivable," as well as non-viable microbes (Bergholz et al., 2014).

**Table 1:** An non-exhaustive list of the mechanistic model-based vs. ML tools for microbial genome analysis.

**Category**	**Tools**	
	**Mechanistic model-based**	**Machine learning**
***DE NOVO*** **GENOME ASSEMBLY**		
	Velvet (Zerbino and Birney, 2008), IDBA-UD (Peng et al., 2012), RAY (Boisvert et al., 2010), SPAdes (Bankevich et al., 2012), SKESA (Souvorov et al., 2018) Minimap/miniasm (Li, 2016), Canu (Koren et al., 2017), REAGO^**^ (Yuan et al., 2015)	PERGA (Zhu et al., 2014), Minimus/AMOS (Palmer et al., 2010), MetaVelvet-SL^*^ (Cheng, 2015)
**GENOME CHARACTERIZATION**		
1. Bacterial strain identification	BLASTN (McGinnis and Madden, 2004), JSpeciesWS (Richter et al., 2016), ANItools (Han et al., 2016), OrthoANI (Lee et al., 2016), KmerFinder (Hasman et al., 2014), StrainSeeker (Roosaare et al., 2017), MESH (Ondov et al., 2016), Kraken^*^ (Wood and Salzberg, 2014), MetaPhlAn^*^ (Segata et al., 2012), QIIME2^**^ (Caporaso et al., 2010), MOTHUR^**^ (Schloss et al., 2009), MG-RAST^**^ (Meyer et al., 2008)	PaPrBaG (Deneke et al., 2017), NBC (Rosen et al., 2008), TACOA (Diaz et al., 2009), PhyloPythiaS+^*^ (McHardy et al., 2007; Gregor et al., 2016), BLCA^**^ (Gao et al., 2017), 16S Classifier^**^ (Chaudhary et al., 2015)
2. Bacterial genome annotation	PROKKA (Seemann, 2014), RAST/myRAST (Overbeek et al., 2014), MetaGeneAnnotator^*^ (Noguchi et al., 2008), MetaGene^*^ (Noguchi et al., 2006), Tax4Fun^**^ (Aßhauer et al., 2015)	Woods (Sharma et al., 2015), Orphelia^*^ (Hoff et al., 2009), MGC^*^ (El Allali and Rose, 2013), MetaGUN^*^ (Liu et al., 2013), Meta-MFDL^*^ (Chen et al., 2016)
3. Virulence gene detection	VirulenceFinder (Joensen et al., 2014), PathogenFinder (Cosentino et al., 2013)	BacFier (Iraola et al., 2012), PaPrBaG (Deneke et al., 2017)
4. Antimicrobial resistance gene detection	ResFinder (Zankari et al., 2012), RGI/CARD (Jia et al., 2017), AMRFinder (Feldgarden et al., 2019)	DeepARG (Arango-Argoty et al., 2018), PATRIC (Antonopoulos et al., 2017)
**COMPARATIVE GENOMICS**		
1. Reference-based SNP methods	CSI Phylogeny (Kaas et al., 2014), Lyve-SET (Katz et al., 2017), CFSAN SNP Pipeline (Davis et al., 2015), SPANDx (Sarovich and Price, 2014), SNVPhyl (Petkau et al., 2017)	
2. Non-reference-based SNP analysis	KSNP (Gardner et al., 2015)	
3. Pangenome-based analysis	Roary (Page et al., 2015), PanWeb (Pantoja et al., 2017), Pan-Seq (Laing et al., 2010)	
4. Core genome/whole-genome multi-locus sequence typing (MLST)	EnteroBase (Alikhan et al., 2018), BIGSdb (Jolley and Maiden, 2010), chewBBACA (Silva et al., 2018)	BAPS/hierBAPS (Cheng et al., 2011, 2013)
**PHYLOGENOMICS**		
	RAxML (Stamatakis et al., 2005), FastTree (Price et al., 2009), CSI Phylogeny (Kanamori et al., 2015), Lyve-SET (Katz et al., 2017), PHYLIP (Shimada and Nishida, 2017), BEAST (Drummond and Rambaut, 2007)	

* The tool is dedicated to shotgun metagenomics;

** *the tool dedicated to 16S metataxonomics*.

## 2. Machine Learning for *de novo* Microbial Genome Assembly

Genome assembly tools are applied with the purpose of assembling the sequencing reads into larger fragments (i.e., contigs), from which near-complete genomes can be further re-constructed. As the read lengths of the second generation (e.g., Illumina) technologies are short (i.e., 50–300 bp), *de novo* assembly without a reference genome remains a challenging task (Zhu et al., 2014). However, *de novo* assembly is especially relevant in FBD outbreak investigations, where the source strain might be undetectable with conventional methods and thus taxonomically unclassified (Quainoo et al., 2017). Currently, the majority of the algorithms are based on the de Bruijn graph or overlap-layout strategies. The de Bruijn graph algorithm first splits up each read into smaller substrings, k-mers, which are further used to construct a graph, in which k-mers represent nodes; two nodes are connected with an edge if they overlap by k-1 nucleotides and follow each other in the read. Thus, each contig is represented as a path within the graph (Zhu et al., 2014). The overlap-layout-based algorithms start by computing the overlaps among all the reads, which are then used to perform the genome assembly (Zhu et al., 2014). For short Illumina read-based single genome WGS, the most popular assemblers include Velvet (Zerbino and Birney, 2008), IDBA-UD (Peng et al., 2012), RAY (Boisvert et al., 2010), SPAdes (Bankevich et al., 2012), and SKESA (Souvorov et al., 2018), all of which employ the de Bruijn graph-based assembly strategy. The overlap-layout-based algorithms are mainly used for the assembly of ultra-long reads: Minimap/miniasm (Li, 2016) and Canu (Koren et al., 2017).

For 16S metataxonomics data, interestingly, there is a tool REAGO (REconstruct 16S ribosomal RNA Genes from metagenOmic data), which combines homology search that considers also the secondary structure and properties of 16S ribosomal RNA genes to perform their *de novo* reconstruction (Yuan et al., 2015).

ML has been used in PERGA (Paired-End Reads Guided Assembler) (Zhu et al., 2014) to determine the correct contig extension. For this, the alogirthm constructs a decision model, considers the avaialble information from paired-end reads such as different read overlap size and various branch features, i.e., path weight, read coverage levels and gap size. In addition, PERGA also detects tandem repeats with the aim to resolve branches in the assembly graph and construct longer and more accurate contigs and scaffolds (Zhu et al., 2014). Minimus/AMOS (Palmer et al., 2010) contains a module that uses ML (C4.5 decision tree, NB and RF) in combination with features identified from prior sequencing projects and completed genomes to classify overlaps as true or false, by this improving the quality of the genome assembly.

For shotgun metagenomics, ML-based strategies has been proposed in order to pre-allocate (i.e., cluster) reads into similar groups before the assembly step, thus reducing the overall computational complexity of the process (Cheng, 2015). Moreover, when assembling metagenomics data, the de Bruijn graph is usually decomposed into individual sub-graphs to build an isolated genome; however, there are still the so called chimeric nodes, i.e., those present in more than one sub-graph, which need to be identified and split apart (Afiahayati et al., 2015). For this, ML (SVM) has been applied, e.g., as implemented in MetaVelvet-SL (Afiahayati et al., 2015).

## 3. Machine Learning for Microbial Genome Characterization

After assembly, the bacterial identity of the isolate usually needs to be identified, followed by genome annotation and identification of those genes that might be of clinical importance, such as antimicrobial resistance and virulence genes. For this, genome characterization tools are being developed which compare the assembled contigs to several reference databases of known genes and reference genomes (Quainoo et al., 2017).

### 3.1. Bacterial Strain Identification

In this category, computational tools, which can assess bacterial identity either directly from reads or from pre-assebled contigs are used (Quainoo et al., 2017). Current tools are often based on genome-wide sequence similarity statistics (Ciufo et al., 2018). NCBI BLAST (the Basic Local Alignment Search Tool) is one of the most popular alignment tools and its variant BLASTN can be used to identify species from contigs using the Nucleotide Collection (nr/nt) database, which contains all the microbial sequences from the NCBI database (McGinnis and Madden, 2004). However, for large-scale read mapping, BLAST may be too slow (Deneke et al., 2017). Generally, this approach may fail to detect novel species in cases when closely related genomes are not found in the reference databases (Deneke et al., 2017), which are known to be biased toward cultivable pathogenic bacteria (Farrell et al., 2018). Average Nucleotide Identity (ANI) (Clingenpeel et al., 2015) has been recently proposed as an alternative metrics for the identification and classification of bacterial species, calculated by performing several pair-wise comparisons of all sequences shared between two given strains. This method is implemented within tools such as JSpeciesWS (Richter et al., 2016), ANItools (Han et al., 2016), and OrthoANI (Lee et al., 2016). Alternatively, composition-based methods such as KmerFinder (Hasman et al., 2014) exist, which employ a precomputed database compiled using 1,647 complete bacterial genomes from the NCBI database divided into 16-mers. Given an input file of unknown bacterial species, the program provides an overview of all k-mers that match all the templates in the database (i.e., the “standard” method) or counts all the k-mers that might originate from a particular strain (i.e., the “winner takes it all” method; Hasman et al., 2014). StrainSeeker (Roosaare et al., 2017) starts with a Newick-format tree and derives a list of k-mers for each node in that tree. Thereafter, the observed vs. expected fractions of node-specific k-mers are being analyzed to determine each node's presence in the input data (Roosaare et al., 2017). MESH (Ondov et al., 2016) is another k-mer based strain identification algorithm that extends the MinHash dimensionality-reduction technique by reducing large (sets of) sequences into small, representative sketches, which are then used to infer global mutation distances.

For shotgun metagenomics, Kraken (Wood and Salzberg, 2014) is a k-mer based approach, which tries to match 31-mers from the input data to a pre-computed database, by considering all reference genomes in which they occur and then mapping these 31-mers to the lowest common ancestor. MetaPhlAn (Segata et al., 2012) first collects all clade-specific marker genes, i.e., from strain to phylum, into a database, which it then utilized for the taxonomic classification of metagenomic shotgun data.

For 16S metataxonomics data, sequence alignment-based approaches are usually used to assign taxa (Chaudhary et al., 2015). For this, QIIME2 (Caporaso et al., 2010), MOTHUR (Schloss et al., 2009), and MG-RAST (Meyer et al., 2008) are the most commonly used pipelines. Overall, the major limitations of the above approaches are the computational time requirements and dependence on the reference databases (Chaudhary et al., 2015).

To overcome these limitations, ML-based approaches have been proposed. NBC (Rosen et al., 2008) calculates k-mer frequency profiles of all publicly available microbial reference genomes and uses these profiles to train a naive Bayesian classifier to identify the respective genome by any query fragment. TACOA (Diaz et al., 2009) achieves taxonomic classification by combining the k-nearest neighbor algorithms with kernel-based ML strategies. Yet another ML-based approach, PaPrBaG (Pathogenicity Prediction for Bacterial Genomes), has been recently proposed, which, in addition to taxonomic classification, also aims to predict the pathogenic potential of the respective strains (Deneke et al., 2017).

For shotgun metagenomics, PhyloPythiaS+ (McHardy et al., 2007; Gregor et al., 2016) is a sequence composition-based method that uses hierarchical structured-output by employing a multiclass support vector machine (SVM) classifier.

For 16S metataxonomics data, prediction-based ML approaches for taxonomic classification have started to emerge, as opposed to homology-based methods (Chaudhary et al., 2015). For example, BLCA is a tool for taxonomic classification of 16S rRNA gene sequences, which combines sequence similarity to the reference database with Bayesian posterior probabilities to weight the degree of sequence similarity of the query sequence to every hit from the database (Gao et al., 2017). 16S Classifier is a similar tool that deploys RF and is compatible with the QIIME2 pipeline (Chaudhary et al., 2015).

### 3.2. Bacterial Genome Annotation

Bacterial genome annotation tools explore which genes are contained in the respective bacterial genome by retrieving the relevant features (i.e., coding regions and their putative products, non-coding RNAs and signal peptides) from raw reads or pre-assembled contigs (Seemann, 2014; Quainoo et al., 2017). PROKKA (Seemann, 2014) is a software suite unifying several feature prediction tools, such as Prodigal (Hyatt et al., 2010) for the identification of coding sequences, RNAmmer (Lagesen et al., 2007), Aragorn (Laslett and Canback, 2004), and Infernal (Kolbe and Eddy, 2011) for the prediction of ribosomal, transfer and non-coding RNA genes, respectively, as well as SignalP (Petersen et al., 2011) to identify signal leader peptides. RAST/myRAST (Overbeek et al., 2014) is another popular genome annotation tool, which uses a SEED k-mer-based annotation algorithm to predict coding sequences, as well as tRNAs and rRNAs.

For shotgun metagenomics, there are several model-based approaches, including MetaGeneAnnotator (Noguchi et al., 2008) or MetaGene (Noguchi et al., 2006), both using Markov chain models to identify genes.

However, the main limitation of these models is that they require optimization of thousands of parameters, which limits their practical use (Zhang et al., 2017). Sequence similarity-based methods, on the other hand, are considered rather time-consuming and computationally demanding, especially when applied to shotgun metagenomic data. This poses a bottleneck for efficient sequencing data analysis (Sharma et al., 2015). Moreover, RAST is known to have difficulties dealing with mixed or contaminated cultures, as its algorithm relies on closely related isolates (Quainoo et al., 2017). In addition, these methods are used to find genes with previously known homologous proteins and cannot predict novel genes (Zhang et al., 2017).

Unfortunately, 16S metataxonomic data does not provide any information on functional genes and proteins for the microbial communities being analyzed (Aßhauer et al., 2015); however, these can be predicted using pangenome-based approaches such as Tax4Fun (Aßhauer et al., 2015).

Alternatively, ML (RF) and similarity-based (RAPsearch2) approaches have been combined in a tool called “Woods” (Sharma et al., 2015); however, it is currently restricted to the prediction of protein coding sequences only.

For shotgun metagenomics, several ML-based methods have been proposed, such as Orphelia (Hoff et al., 2009), MGC (El Allali and Rose, 2013), MetaGUN (Liu et al., 2013), and Meta-MFDL (Zhang et al., 2017), e.g., the latter using a deep stacking networks learning model and multiple genomic features (i.e., the usage of monocodons and monoamino acids) for identifying genes from metagenomic fragments (Zhang et al., 2017).

### 3.3. Virulence Gene Detection

In this part of the analysis, the aim is to explore whether the previously annotated genes infer virulence, i.e., some degree of pathogenicity to the host (Quainoo et al., 2017). However, virulence gene detection does not necessarily have to follow the genome annotation step. It can also be performed either using reference database entries as BLAST queries against assembled genomes or mapping raw reads against reference database entries (or any other collection of genes of interest). Also, predicted (but not annotated) coding DNA (or predicted protein) sequences can be screened for virulence gene content. The most commonly used reference database for virulence genes is the Virulence Factor Database (VFDB) (Chen et al., 2016), containing information on 951 bacterial strains and 1,075 virulence factors (as of March 2019), including different characteristics, such as whether a virulence factor is used in offensive or defensive actions. Recently, VFDB has been supplemented with VFanalyzer, a Web-based tool that builds orthologous groups of genes using a query genome and pre-analyzed reference genomes and then performs sequence similarity searches among the VFDB gene collection for atypical and strain-specific virulence genes (https://doi.org/10.1093/nar/gky1080). Frequently used tools to predict virulence genes from sequencing data include VirulenceFinder (Joensen et al., 2014), a Web-based tool that uses BLASTN (Camacho et al., 2009) and contains virulence markers for four microbes: *Listeria, S. aureus, E. coli*, and *Enterococcus*. Another Web-based tool is PathogenFinder (Cosentino et al., 2013), which assumes that bacterial pathogenicity (or lack of it) depends on groups of proteins that are consistently found together in either pathogens or non-pathogens. PathogenFinder aims to identify such groups of proteins.

Several ML-based approaches have been proposed for virulence gene detection. VirulentPred (Garg and Gupta, 2008) is a bi-layer cascade SVM-based prediction method, where the first layer classifiers are being trained using different protein sequence features, such as amino acid and dipeptide composition. The results from the first layer are then passed to the second layer classifier, which utilizes sequence similarity and a BLAST database containing both virulence and non-virulence genes. BacFier (Iraola et al., 2012) uses known pathogenic vs. non-pathogenic strains and their genetic features (e.g., the presence or absence of different virulence-related genes) to train ML algorithms in predicting pathogenicity of input bacterial genomes. Finally, as described above, PaPrBaG (Deneke et al., 2017) also aims to predict the pathogenic potential of microbial strains by means of training on a large number of established pathogenic species in comparison with non-pathogenic bacteria and their sequence features. PaPrBaG is a RF-based method for the assessment of the pathogenic potential of a set of reads belonging to a single genome. It helps in the prediction of novel, unknown bacterial pathogens. PaPrBaG provides prediction in contrast with other approaches that discard many sequencing reads based on the low similarity to known reference genomes.

### 3.4. Antimicrobial Resistance Gene Detection

In this step, computational analysis is used to explore whether the previously annotated bacterial genes infer antimicrobial resistance, i.e., the ability of microorganisms to grow despite exposure to antimicrobial substances (Quainoo et al., 2017). However, again, the same is true as for virulence gene prediction—this step does not necessarily have to follow the genome annotation step, e.g., it can be also conducted right after assembly. Frequently used tools for this purpose include a Web-based tool ResFinder (Zankari et al., 2012) and RGI/CARD (Jia et al., 2017). Both perform homology-based resistome prediction: ResFinder (Zankari et al., 2012) uses BLAST, whereas RGI/CARD (Jia et al., 2017) makes use of a manually curated resource containing antimicrobial resistance genes, proteins and mutated sequences—CARD (Jia et al., 2017). Resently, NCBI has developed AMRFinder (Feldgarden et al., 2019) which utilizes the NCBI's curated AMR gene database - Bacterial Antimicrobial Resistance Reference Gene Database-, currently including 4,579 antimicrobial resistance gene proteins and over 560 hidden Markov models (HMMs).

ML approaches for the same task include DeepARG (Arango-Argoty et al., 2018), a deep learning approach using neural networks and previously curated databases, such as CARD (Jia et al., 2017), for predicting antibiotic resistance genes and annotating them to 30 known antibiotic resistance categories, creating a manually curated database, DeepARG-DB. PATRIC (Antonopoulos et al., 2017) uses the genomes in its in-house database and their antimicrobial resistance-related metadata, such as susceptibility or resistance to a given antibiotic, to build AdaBoost (adaptive boosting) ML-based classifiers and predict those regions within a bacterial genome that are associated with antimicrobial resistance (Davis et al., 2016). When a genome is submitted to the PATRIC annotation service, these classifiers are used to predict if the organism is susceptible or resistant to an antibiotic. However, PATRIC is limited to identifying only genes encoding resistance to certain antibiotics (beta lactam, carbapenem, and methicillin) and in certain bacterial species. In this context, ML has also been applied to identify genomic features possibly related to minimum inhibitory concentration (MIC) of an antibiotic, i.e., its lowest concentration preventing visible growth of bacterium *in vitro*, e.g., for Nontyphoidal *Salmonella* (Nguyen et al., 2019).

## 4. Machine Learning for Microbial Comparative Genomics

After characterization of an individual genome is accomplished, the next step is to perform comparative genomics and detect relatedness between strains, identify potentially clonal strains and pinpoint the putative source of the outbreak (Brown et al., 2019). Bacterial species should be determined before performing comparative genomic analyses, since most algorithms will perform better when closely related bacterial strains can be used. Comparative genomics methods can be largely divided into three groups: (i) reference/non-reference-based SNP-based methods, (ii) pangenome-based and (iii) core genome/whole-genome multilocus sequence typing (MLST).

### 4.1. Reference-Based SNP Methods

Standard strategies to identify genetic variation, which occurs in a strain, usually focus on single nucleotide polymorphisms (SNPs). Raw reads are mapped to a perform better when closely related, high-quality reference genome, identifying SNPs as variations in relation to that reference genome. CSI Phylogeny (Kaas et al., 2014), Lyve-SET (Katz et al., 2017), CFSAN SNP Pipeline (Davis et al., 2015), SPANDx (Sarovich and Price, 2014), and SNVPhyl (Petkau et al., 2017) include such pipelines. In addition, there are also tools such as Harvest/Parsnp (Treangen et al., 2014) that, instead of trying to performing whole-genome alignment, focus on constructing a core-genome alignment, i.e., identifying a set of orthologous sequence conserved in all aligned genomes. However, reference-based SNP methods are generally recommended only if a high-quality reference genome exists (Brown et al., 2019), when higher resolution is required than can be achieved using cgMLST/wgMLST, or when a cgMLST/wgMLST scheme is not available (Katz et al., 2017).

### 4.2. Reference-Free SNP Analysis

Reference-Free SNP Analysis does not require alignment to a reference genome to identify SNPs. Such examples include kSNP (Gardner et al., 2015), a k-mer-based approach where the user provides the length of the flanking sequence including the SNP, i.e., the SNP is at the central base of the k-mer, and the flanking (k-1)/2 bases on both sides of the SNP define the locus. First, kSNP counts all k-mer oligos for each input genome. This is followed by several filtering steps: (i) the k-mer list is then condensed so that counts reflect both occurrences on the forward and reverse strands; (ii) for raw reads, kSNP discards k-mers that occur only once, as such singletons are likely to be sequencing errors; (iii) for each genome, kSNP discards k-mers that have more than one central base variant for a given locus. Finally, kSNP merges and sorts all k-mers across all user provided genomes and looks for SNP loci in the merged list. Then it compares the SNP loci for each genome with the merged list to identify the SNPs in each genome, reporting the locus and the central base, i.e., the SNP, for every genome containing that locus (Gardner et al., 2015).

### 4.3. Pangenome-Based Analysis

Pangenome-based analysis classifies genes as the so called core genes, found in all bacterial strains under comparison, and into accessory genes that can be found only in several but not all strains (Page et al., 2015). Isolates are then clustered based on their accessory genome (Page et al., 2015). A well-known tool for pangenome-based analysis is Roary (Page et al., 2015). First, it identifies orthologous genes by sequence comparison. This is followed by grouping of these genes into clusters. Finally, the relationships of the clusters are then represented using a graph, constructed based on the order in which their occur in the input data (Page et al., 2015; Brown et al., 2019). Other tools for pangenome-based analysis include PanWeb (Pantoja et al., 2017) and Pan-Seq (Laing et al., 2010).

### 4.4. Core Genome/Whole-Genome Multi-locus Sequence Typing (MLST)

Core genome/whole-genome multi-locus sequence typing (MLST) are widely used methods for outbreak investigations, enabling standardized outbreak management protocols (Nadon et al., 2017; Brown et al., 2019). Conventional MLST usually uses only seven genes/loci to derive sequence types (STs), and is not always able to distinguish between outbreaks resulting from closely related bacterial variants (Pearce et al., 2018). Core genome MLST (cgMLST) schemes extend the conventional MLST, including genes/loci present in 95% to 99% of isolates, hence offering increased resolution to detect isolate-specific genotypes, as well as novel transmission events (Nadon et al., 2017; Brown et al., 2019). If two strains display identical cgMLST profiles, these are being grouped into one cluster type (CT), which can be shared using dedicated databases (Quainoo et al., 2017). CgMLST is implemented within the Ridom SeqSphere+ commercial software suite (JÃijnemann et al., 2013). However, it is also being utilized by EnteroBase (Alikhan et al., 2018), Bacterial Isolate Genome Sequence Database (BIGSdb) (Jolley and Maiden, 2010) and chewBBACA (Silva et al., 2018). On the other hand, whole-genome MLST (wgMLST) further extends cgMLST, as it also considers the accessory genes to detect lineage-specific loci. This method is part of the BioNumerics (Applied Maths) software suite since version 7.5 (http://www.applied-maths.com/) and is also implemented within EnteroBase (Alikhan et al., 2018). For outbreak investigations, cgMLST is more suited, as it uses species-specific nomenclature; however, wgMLST might offer higher resolution to discriminate outbreak strains that form closely related clusters (Nadon et al., 2017; Brown et al., 2019). Of note, however, both methods strongly depend on the availability of high-resolution isolate typing schemes (Pearce et al., 2018), which may not be available for lesser-studied foodborne pathogens, due to the lack of publicly available WGS data (Carroll et al., 2019).

To the best of our knowledge, ML-based tools do not seem to have gained a lot of attention in comparative genomics. The Bayesian Analysis of Population Structure (BAPS)/hierBAPS (Cheng et al., 2011, 2013) tool seems to be the only ML-based tool for comparative genomics. BAPS/hierBAPS was created by first collecting large data sets of multi-locus DNA sequence types (STs), as well as the respective metadata (e.g., host organism, serotype) from several MLST databases PubMLST (http://www.pubmlst.org). This data was then utilized to divide the available pathogens into subsets of different evolutionary lineages or geographically related sub-populations, as determined based on molecular [dis]similarities within the database. Then a user-submitted set of bacterial isolates can be classified to one of these groups, using a Bayesian model-based ML algorithm. In addition, recently, several other studies have combined comparative genomics with ML approaches for the classification of outbreak strains (Diaz et al., 2017) or source tracking during outbreaks (Buultjens et al., 2017; Zhang et al., 2019). Diaz et al. (2017) identified six distinct subtypes of genomes, as well as their respective SNPs/loci, and trained RF to separate input genomes into the respective subtypes. Buultjens et al. (2017) used core genome variation and classification based on principal components to identify genomic signatures specific to source of interest, which were further used to predict the origin of input isolates (Buultjens et al., 2017). Zhang et al. (2019) used a set of genetic features extracted from *Salmonella* Typhimurium genomes, inlcuding core genome SNPs, insertion/deletions and accessory genes to train a RF classifier in discriminating isolates from swine, bovine, poultry or wild bird sources. Wheeler et al. (2018) investigated genomic signatures related to host adaptation in *Salmonella enterica*. First, hidden Markov models were used to identify patterns of sequence variation and their potential functional consequences. Thereafter, RF was utilized to identify genes that displayed differences between lineages with different phenotypes (Wheeler et al., 2018). Sharma et al. (2014) used MLST to differentiate isolates and categorize an unknown isolate as either representing a true infection or a likely contaminant. In particular, the seven genotypes derived from MLST were used to train three different ML algorithms (SVM; Classification And Regression Tree Analysis - CART; and a Naive Nearest-Neighbor Classifier) to segregate isolates of known class (i.e., pathogen or likely contaminant) on the basis of their alleles, which were then used to classify an unknown isolate by its MLST allele profile.

## 5. Machine Learning for the Inference of Microbial Phylogenomics

Finally, comparison tools can be used for the inference of microbial phylogenomics of pathogenic isolates and generate detailed networks reflecting the transmission events of outbreak strains between different patients (Quainoo et al., 2017). In particular, phylogenomics can reveal whether two isolates are nearly identical or only distantly related and which might represent the initial outbreak source strain (Quainoo et al., 2017). Maximum likelihood is frequently applied when characterizing pathogens from foodborne outbreaks. RAxML (Randomized Axelerated Maximum Likelihood) (Stamatakis et al., 2005) and FastTree (Price et al., 2009) are two maximum likelihood based phylogenomics estimators, which work by first constructing an initial tree, which is then further refined in several optimization steps and tree rearrangements to increase the likelihood that the respective tree reflects the evolutionary relationships of the input sequences. These software packages are often included in the genome comparison pipelines mentioned in the previous chapter such as CSI Phylogeny (Kaas et al., 2014) and Lyve-SET (Katz et al., 2017) for streamlined production of actionable results. Alternatively, distance matrix-based methods such as neighbor joining (Saitou and Nei, 1987) (e.g., part of the PHYLIP Shimada and Nishida, 2017 package) as well as Bayesian analysis-based methods (e.g., BEAST Drummond and Rambaut, 2007) have been proposed to study microbial phylogenomics.

Most recently, Suvorov et al. (2019) has proposed an approach that uses convolutional neural networks (CNNs) for phylogenetic inference. In particular, CNNs are being trained to extract phylogenetic signal from a multiple sequence alignment, which is then used to reconstruct and discriminate alternative tree topologies. Of note, however, this study used an alignment of only four sequences.

## 6. Conclusions

Over the last years, several ML-based tools have been developed for different steps of bacterial WGS analysis. However, some areas of bacterial bioinformatics (i.e., genome assembly and strain identification) have seen more development than others (i.e., phylogeny estimation). Overall, AI and its sub-discipline ML could lead to actionable knowledge in diverse ranges of sectors, where multiple complex challenges need to be addressed, including the outbreak investigations of foodborne pathogens and antimicrobial resistance (Gibbs, 2014; Quainoo et al., 2017; Ching et al., 2018), considering that WGS may replace conventional analysis methods already in the near future (Quainoo et al., 2017). In this scenario, the success of outbreak investigations will largely depend on how fast and accurate WGS data can be produced and analyzed (Quainoo et al., 2017). ML-based algorithms could further speed-up such investigations, especially as the number of complete microbial genomes in NCBI RefSeq (http://www.ncbi.nlm.nih.gov/genome) is rapidly growing (Tatusova et al., 2015), providing a valuable resource for training ML classifiers. However, even if substantially improving the accuracy and speed of WGS algorithms, a number of limitations still need to be overcome in order to fully utilize the power of ML for outbreak screenings. WGS analysis tools often rely on sequence similarity and hence strongly depend on reference databases (Deneke et al., 2017; Zhang et al., 2017). Moreover, such methods are rather time-consuming and computationally demanding, thus representing a bottleneck for efficient sequence data analysis (Sharma et al., 2015). ML algorithms could potentially increase the accuracy and speed of clinically and epidemiologically relevant predictions (Farrell et al., 2018). However, to yield accurate predictions, besides the choice of the most appropriate algorithm and a set of well-defined inputs and outputs of interest, ML-based strategies generally require large amounts of high-quality training data (Baker et al., 2018). This presents a limitation, as currently microbial genome databases are known to be biased toward cultivable pathogenic bacteria. The current lack of large and comprehensive databases can be considered as the key bottleneck for the application of ML methods (Farrell et al., 2018). Hence, future improvements can be expected to come from better data curation and collection, in addition to development of new and improved classification algorithms (Farrell et al., 2018). Therefore, WGS data collection must be done in parallel with comprehensive and standartized metadata collection such as phenotypic profiling using traditional microbiology methods for isolate characterization (e.g., phenotypic profiling of antimicrobial resistance) (Maurer et al., 2017).

Currently, sequencing of bacterial genomes is mostly performed on Illumina instruments, producing relatively short reads with limited resolution of low-complexity regions (Quainoo et al., 2017). Alternatively, ultra-long read technologies such as ONT (https://nanoporetech.com/) and PacBio SMRT (https://www.pacb.com/smrt-science/smrt-sequencing/) are increasingly being used to obtain complete microbial genomes. However, both technologies are still three and almost seven times more expensive in comparison to Illumina short-read sequencing (Brown et al., 2017; Sekse et al., 2017; Nicola De Maio, 2019). Moreover, both technologies still display rather high error rates (Mahmoud et al., 2017), which makes them more suitable for gap closure in draft genomes using hybrid methods (Quainoo et al., 2017). Hence, error-profile-aware ML-algorithms implementing hybrid strategies that make use of more accurate short reads in conjunction with ultra-long reads may need to be considered for future applications.

The selection of a harmonized bioinformatics strategy or pipeline that would perform consistently across outbreak investigation situations around the world, reaching consensus on desired standards represents another challenge for the routine implementation of WGS analysis (Quainoo et al., 2017). Especially, considering that the numbers of commercial analysis software platforms, as well as open-source, application-specific analysis tools are increasing, a rigorous assessment and benchmarking of their quality is urgently needed (Quainoo et al., 2017). This would also be a prerequisite for a systematic comparison between ML-based vs. conventional methods. Nevertheless, in order to perform such comparisons on a global scale, WGS data storage and sharing would be of utmost importance. Although technically feasible, this will require us to solve several issues of ownership and data privacy, making sure that these are being adequately protected (Quainoo et al., 2017).

## Author Contributions

BV wrote the manuscript. IM, LG-I, and JK participated in revising and editing the manuscript. All authors have read and approved the final version of the manuscript.

### Conflict of Interest Statement

BV is the CEO of net-OMICS, a bioinformatics company. The remaining authors declare that the research was conducted in the absence of any commercial or financial relationships that could be construed as a potential conflict of interest.
